# Frequency of D222G haemagglutinin mutant of pandemic (H1N1) pdm09 influenza virus in Tunisia between 2009 and 2011

**DOI:** 10.1186/1746-1596-8-124

**Published:** 2013-07-31

**Authors:** Awatef El Moussi, Mohamed Ali Ben Hadj Kacem, Francisco Pozo, Juan Ledesma, Maria Teresa Cuevas, Inmaculada Casas, Amine Slim

**Affiliations:** 1 National Influenza Centre-Tunis, Unit Virology, Microbiology Laboratory, Charles Nicolle’s Hospital, Tunis, Tunisia; 2 National Influenza Centre-Madrid, Influenza and Respiratory Viruses Laboratory, Instituto de Salud Carlos III, Madrid, Spain

**Keywords:** D222G substitution, Haemagglutinin, Influenza A(H1N1)pdm09 virus, Pandemic, Severe respiratory infection

## Abstract

**Background:**

The novel pandemic A (H1N1) pdm09 virus was first identified in Mexico in April 2009 and since then it spread worldwide over a short period of time. Although the virus infection is generally associated with mild disease and a relatively low mortality, it is projected that mutations in specific regions of the viral genome, especially within the receptor binding domain of the haemagglutinin (HA) protein could result in more virulent virus stains, leading to a more severe pathogenicity.

**Methods:**

To monitor the genetic polymorphisms at position 222 of Haemagglutinin of influenza A(H1N1)pdm09 viruses from both outpatients with mild influenza and individuals with severe disease requiring hospitalization, during 2009–2010 and 2010–2011 seasons, a sequence-based genotypic assessment of viral populations to understand the prevalence of D222G mutation.

**Results:**

The D222G was identified in clinical specimens from 3 out of 42 cases analyzed in Tunisia with severe outcome (7%). Interestingly, in one fatal case out of four viruses taken from fatal cases studied (25%). Also this mutation was found in one mild case out of 8 mild cases studied (0.1%). D222E substitution was found in virus taken from one patient with severe clinical syndrome (2%) out of 42 severe cases analyzed and E374K substitution was found in two severe cases (4%) out of 42 severe cases studied.

**Conclusions:**

A specific mutation in the viral haemagglutinin (D222G) was found in fatal, severe and mild case. Further virological, clinical and epidemiological investigations are needed to ascertain the role of this and other mutations that may alter the virulence and transmissibility of the pandemic influenza A (H1N1)pdm09.

**Virtual Slides:**

The virtual slide(s) for this article can be found here: http://www.diagnosticpathology.diagnomx.eu/vs/1027334947811255

## Introduction

In April 2009, a novel swine-derived influenza A(H1N1)pdm09 emerged and spread rapidly around the world [1,2], causing the World Health Organization to declare a pandemic in June. Since the first appearance of influenza A(H1N1)pdm09, one particular amino acid substitution {aspartic acid to glycine at position 222 (D222G)} (225 in H3 numbering) within the hemagglutinin (HA) molecule has appeared sporadically in 20 countries, including Norway, Mexico, Ukraine and the USA [3-5]. The D222G substitution is known to cause a shift from α2,6-SA receptor specificity to mixed α2,3/α2,6-sialic acid receptor specificity [6,7]. It is noteworthy that is highly conserved among avian viruses [8]. Previously, α2,3-specific avian viruses have been isolated from patients during the initial phases of the pandemics of 1957 and 1968, and avian HA in humans has been shown to be selected for increased affinity for the α2,6 receptor [8]. Also, the substitution was present in the Spanish Flu outbreak of 1918 [9]; however, the existence and transmissibility of influenza A(H1N1)pdm09 α2,3-SA specific viruses remain unclear. To identify whether α2,3-SA specific viruses, which replicate well in swine, were spread during the early phase of the pandemic and whether α2,3-SA specific viruses are easily transmitted, the nucleotide sequences of the HA receptor binding site of influenza A(H1N1)pdm09 in clinical specimens were determined in this study.

In an attempt to understand the relevance of HA D222G substitution among influenza A(H1N1)pdm09 causing infections in Tunisia, HA gene sequences from respiratory specimens of severe and non-severe cases were examined. In addition to the D222G substitution, we focused on another substitution {glutamic acid 374 to lysine acid (E374K)}, mutation located at the stalk of HA2 in the cavity where the fusion domain of mature HA molecules might have an impact on the antigenicity or neutralization activity of influenza A(H1N1)pdm09 [10-12].

## Material and methods

Nasopharyngeal or throat swab specimens from influenza patients are received directly from sentinel primary care physicians participating in virological surveillance schemes in the community. Samples are also received from community, hospitalised and fatal cases are forwarded to the Tunisia National Influenza Centre for diagnostic and further characterisation. A total of 7350 specimens from influenza patients were collected in Tunisia during 2009–2010 season and 894 specimens during 2010–2011 season.

The samples used in this study were taken from 50 patients including 42 respiratory specimens from severe (patients hospitalized with severe pneumonia and severe acute respiratory syndrome) and fatal cases, as well as from 8 cases with mild clinical outcomes. Mild cases presented with at least one of the following influenza-like illness symptoms: fever of at least 38°C, cough, rhinorrhea, headache, or abdominal symptoms (i.e., diarrhea and vomiting).

Viral RNA was extracted from respiratory samples (Oro-pharyngeal and nasopharyngeal swabs) using commercially available QIAamp Viral RNA Mini Kit QIAGEN as per manufacturer’s instructions. For initial detection of Influenza A virus, amplification of matrix protein (M) gene was carried by real time RT-PCR CDC protocol [13]. For subtyping of Influenza A positive samples, the HA gene (segment 4) of influenza A(H1N1)pdm09 viruses were analyzed by specific real-time PCR using “Influenza virus A 1 Real Time RT-PCR Kit” (Shanghai ZJ Bio-Tech Co. Ltd). In order to identify the Changes in HA amino acid diversity in individual cases, genetic characterisation is performed by targeted haemagglutinin (HA) sequence analysis and/or partial genome (931 nucleotide residues) sequencing for a subset of isolates. All viruses analyzed were amplified and sequenced according to the protocol of National Influenza Centre Madrid [14]. Primers PHA1+ (5’-GGGGTTAGCAAAAGCAGGRG-3’) and PHA1− (5’-CAWCCRKCIAYCAKICCWKICCAICC-3’) were used for RT-PCR and H1 + SSEQB (5’-AAYAAYTCIACYGACACTG-3’) and H1-ASEQ (5’-CCCTCAATRAAACCRGCAAT-3’) for nested PCR.

The sequences were analyzed using the maximum composite likelihood method and the MEGA version 4.0 software package with 500 bootstrap replicates [15]. Nucleotide sequence accession numbers: The nucleotide sequences of HA determined in this study can be found in GenBank databases under the indicated accession numbers JN037697 to JN037779 (http://WWW.ncbi.nih.gov/genomes/FLU/SwineFlu.html).

### Ethical approval

The ethical aspects of this study were approved by Charles Nicolle's Hospital ethic committee.

## Results

During the pandemic year, a total of 3836 out of 7350 respiratory specimens with ILI coming from the sentinel physicians network were positive for influenza A(H1N1)pdm (95%). In 2010–2011 season, 146 out of 894 of the cases were positive for influenza A(H1N1)pdm09 (70%). Here we report the occurrence of an amino acid substitution, aspartic acid to glycine in position 222 (D222G) in the HA subunit of the viral haemagglutinin, in clinical specimens from 3 out of 42 cases analyzed in Tunisia with severe outcome (7%). Interestingly, in one fatal case out of four viruses taken from fatal cases studied (25%). This patient died after 3 days, suffering of severe respiratory symptoms of flu. Autopsy revealed pulmonary oedema, large mucosis secretions but no cardiac inflammation (Table 1). Also this mutation was found in one mild case out of 8 mild cases studied (0.1%). Moreover, D222E was found in one out of 50 viruses studied. This mutation was found in virus taken from one patient with severe clinical syndrome out of 42 severe cases studied (2%). E374K substitution was found in two severe cases (4%). This analysis of HA also showed frequent substitutions in other positions. P83S and S203T were detected in 94% of Tunisian viruses studied.

**Table 1:** Characteristics of patients according to the outcome of the infections and molecular analysis

**Virus strains of influenza A(H1N1)pdm09**	**Date of sampling**	**GenBank accession number**	**Mutation**	**Age**	**Sex**	**Clinical information**
A/Tunisia/20043/2009	12/12/2009	JN037731	K374E	33	Male	Severe pneumonia
A/Tunisia/20112/2009	14/12/2009	JN037733	D222E	43	Male	Severe pneumonia with acute respiratory syndrome
A/Tunisia/1064/2010	18/01/2010	HM590676	D222G	47	Male	Severe pneumonia and death
A/Tunisia/197/2011	04/01/2011	CY080589	K374E	46	Female	Severe pneumonia
A/Tunisia/1411/2011	7/02/2011	JN037741	D222G	15	Male	Mild case
A/Tunisia/1701/2011	10/02/2011	JN037743	D222G	45	Male	Severe pneumonia requiring intensive care

D, aspartic acid; G, glycine; E, glutamic acid; K, lysine.

## Discussion

Genetic analysis of HA of influenza A (H1N1)pdm09 virus showed that this virus was a reassortant containing gene segments from ancestor viruses of human, swine and avian sources. Hence, Polymorphism at position 222 within the haemagglutinin (HA) molecule may have remarkable impact on viral host range, replication, and pathogenicity. It is worth noting that although the Asp222Gly mutation currently has not been associated with severe pandemic in humans.

An association between D222G and severity was initially proposed by Kilander et al. (2010) [16] and, since then, different studies in several countries [17] have found the D222G substitution to be more frequently associated with patients with severe pandemic influenza than in non-severe control cases. A recent study supported a role for this mutation in allowing the virus access to deeper lung tissue [18]. Hese unusual viral attributes suggested that this new virus may possess some virulence characteristics similar to the highly pathogenic H5N1 or the 1918 pandemic influenza viruses.

This study used a conventional sequencing approach to analyze 50 H1N1pdm samples obtained from 2009 to 2011. Viruses with D222G substitution in HA protein have appeared sporadically and spontaneously in Tunisia since July 2009 [19]. Although, 7% of them, found in severe cases in the present study, had the D222G substitution. This percentage is comparable to that found in Italy (4%) [5], in United Kingdom (6%) [17], in France (8%) [20] or in Spain (5%) [14], and lower than that published in Norway (18%) [16], and in Hong Kong (17.4%) [21]. These differences with the Norwegian and Chinese study might be due to the reduced number of severe cases analyzed in that country. In Tunisia, this mutation was observed in circulating virus obtained from severe cases [19] and also from mild cases. The frequency of D222G substitution is higher in severe cases than mild cases. Although most of studies demonstrated the presence of D222G substitution in severe cases, it was also reported in mild cases [5,22]. Therefore, the number of mild cases would need to be larger to determine whether mutant viruses are indeed circulating at a very low frequency also in non-severe cases.

The 222 G/E polymorphisms in the haemagglutinin (HA) gene of influenza A(H1N1)pdm09 virus have been associated with cases of mild to severe illness from different countries or geographical areas [23]. Many retrospective analyses have found that cases bearing the D222G mutation were more likely to be associated with severe pneumonia, admission to intensive care facilities, and death [24]. The majority of studies have reported that presence of D222G is sufficient to enhance virus replication and lethality in mouse models, with this effect ranging from modest to pronounced [25,26]. Moreover, D222G substitution had been present in the Tunisian virus strain since pandemic season and throughout 2010–2011 season. Other groups have not observed substantial differences between wild-type and D222G viruses in mouse or ferret models [6], indicating the need for further investigation into the role of D222G in virulence of influenza A (H1N1)pdm09. The D222E was detected with less frequency than the D222G and only found in severe case patient. Despite the available virological, epidemiological and clinical information, the D222E substitution could confer more severity to the disease. The clinical significance of this mutation is still unclear [27]. All above studies demonstrated that polymorphism of the HA protein, especially within the receptor binding domain, play a critical role in the binding preference and pathogenicity of influenza A(H1N1)pdm09 virus. Further study is warranted to elucidate the intriguing relationship between D222G substitution and severe disease.

## Conclusion

Whether the selection of the D222G mutation is a cause or a consequence of more severe lower respiratory tract infection is still to be resolved. It is evident, however, that its emergence is likely to exacerbate the severity of disease. The altered receptor specificity and distinctive cell tropism of the D222G mutants of influenza A(H1N1)pdm09 are hallmarks of a more dangerous pathogen, emphasizing the importance of close monitoring of the evolution of these viruses. Influenza A(H1N1)pdm09 variants with 222 G/E polymorphisms showed increased clinical virulence, and detection of such mutants in the next epidemics is mandatory for better management of ILI in individual patients as well as for surveillance purposes especially in African countries.

## Consent

For all participants, respiratory samples were collected after informed consent, under the supervision of local sanitary authorities.

## Abbreviations

HA: Haemagglutinin; SA α2,6: Sialic acids α2,6; SA α2,3: Sialic acids α2,3; ILI: Influenza-like illness.

## Competing interests

None of the authors has a financial or personal conflict of interest related to this study.

## Authors’ contributions

AEM: proposed the idea, analyzed and interpreted the data presented in the paper. Also she wrote the manuscript; FP and JL: participated in the data analysis and interpretation. Also they revised the manuscript; MTC and IC: revised the manuscript; AS and MHK: revised the manuscript and save final approval of the version to be published. All authors read and approved the final manuscript.
